# Inhibitory effect of Endostar on HIF-1 with upregulation of MHC-I in lung cancer cells

**DOI:** 10.1080/15384047.2025.2508535

**Published:** 2025-05-20

**Authors:** Ming-Zhen Zhao, Hong-Fei Zheng, Jing-Na Wang, Yan-Min Zhang, Hai-Jing Wang, Zhi-Wei Zhao

**Affiliations:** a Hebei Key Laboratory of Panvascular Diseases, Affiliated Hospital of Chengde Medical University, Chengde, Hebei, China; b Department of Pulmonary and Critical Care Medicine, Affiliated Hospital of Chengde Medical University, Chengde, Hebei, China

**Keywords:** Endostar, HIF-1, MHC-I, β2m, lung cancer

## Abstract

Endostar is a human recombinant endostatin which is an attractive anti-angiogenesis protein. Because inefficient antigen presenting MHC class I expression (which can be downregulated by HIF-1) is an important strategy for cancer immune evasion, besides its anti-angiogenesis effect, it remains unclear whether Endostar has an inhibitory effect on HIF-1 expression by upregulating MHC class I expression in cancer cells to facilitate immunotherapies, including PD-1/PD-L1 inhibitors. In this study, A549 and NCI-H1299 lung cancer cells were treated with Endostar (6.25 μg/ml, 12.5 μg/ml, and 25 μg/ml, respectively). HIF-1 expression was detected by Immunocytochemistry and Western blot. Proteins of the MHC class I α-heavy chain and β2 m light chain, STAT3 and pSTAT3 were detected by Western blot. The mRNAs of MHC class I α-heavy chain and β2 m light chain were detected by RT-qPCR. It was shown that decreased expression of HIF-1 and promotion of β2-microglobulin were observed after Endostar treatment. In addition, elevated levels of MHC class I α-heavy chain mRNA and protein, as well as downregulation of STAT3 and pSTAT3, were also observed following Endostar treatment. Endostar inhibited HIF-1 expression in A549 and NCI-H1299 lung cancer cells, upregulated expression of MHC class I α-heavy chain and β2 m light chain, with the upregulation of STAT3 and pSTAT3, suggesting involvement of STAT3 pathway. It is important because only in combination with MHC class I on target cells can tumor antigenic peptides be recognized by CD8+ CTLs which destroy target cells. However, MHC class I is frequently deficient in cancer cells.

## Introduction

For the last several decades, lung cancer has long been the most common cancer worldwide and the leading cause of death from cancer with poor survival and high fatality rate of this disease.^1,2^ The individual risk for development of lung cancer arises from several factors, including smoking and age, radon exposure, asbestos, and other harmful substance exposure, socioeconomic deprivation, earlier diagnosis of a malignant tumor, earlier diagnosis of respiratory conditions, such as chronic obstructive pulmonary disease (COPD), family history, as well as particular rare hereditary disorders, such as Li Fraumeni syndrome and the recently described non-syndromic association with germline EGFR mutation.^3^ Besides, inefficient immunosurveillance, which was caused by immune evasion strategy used by cancer including lung cancer, plays an important role.

Cancer cells can be eliminated by the host immune system because, even though they are self origined, the cancer cells differ from their normal counterparts in terms of their biological behaviors, antigenic characteristics, and biochemical characteristics. Due to an inefficient DNA damage repair system, tumor-specific neoantigens arise and are expressed in cancer cells. The neoantigens bind to the human leukocyte antigen (HLA) system class I, which are the major histocompatibility class I (MHC-I) molecules in humans. The neoantigens binding to HLA-I are presented for CTLs. CTLs eliminate cancer cells through two main mechanisms: direct perforin-dependent destruction and secretion of inflammatory cytokines, such as tumor necrosis factor (TNF) and interferon (IFN) alpha (INF-α), which increase tumor immune sensitivity.^4–6^

Despite the host immune system’s capability）to recognize and destroy cancer cells, the host immune system frequently fails to control cancer growth^7,8^ because cancer cells have acquired the capability to evade recognition and destruction by immune system. Hypoxia is beneficial for malignant tumors to get rid of immune surveillance.^9–11^ In solid tumors, because of uncontrolled growth of cancer cells and disorganized neoangiogenesis, cellular oxygen availability is reduced, leading to intratumoral hypoxic conditions.^12^ In many types of tumor cells, hypoxia-inducible factor 1 (HIF-1) is highly expressed.^13^ HIF-1 signaling pathways play an important role in metabolic adaptation to hypoxia stress.^14–16^ HIF-1 signaling also induces immune checkpoint molecules and immunosuppressive factors to express, which suppresses innate and adaptive immune systems in order to escape from immune attack.^17^ MHC class I molecules, which are necessary for antigen-presentation, are also down-regulated by HIF-1, thus limiting T cells recognizing tumor cells.^18^

Human cancers of different histological origins have shown altered or complete loss of MHC class I, which subsequently affects the final outcome of immunotherapy. Thus, there is a clear need to improve tumor immunogenicity.^19–22^

The high expression of HIF-1 in tumor cells and its role in MHC-I down-regulation make it an important target in cancer immunotherapy. Exploring drugs that inhibit HIF-1 and upregulate MHC-I is an important objective. Endostar may be an important candidate of such drugs. Endostar is a human recombinant endostatin which is an attractive anti-angiogenesis protein. It has been approved by the State Food and Drug Administration of China (CFDA) because of its clinical application on non-small cell lung cancer (NSCLC).^23,24^ Nevertheless, besides its anti-angiogenesis, it remains unclear whether Endostar also inhibits HIF-1 expression by upregulating MHC class I expression in cancer cells, which is helpful for immunotherapies, including PD-1/PD-L1 inhibitor, to induce effective responses of cell-mediated immunity against cancers such as lung cancer. This study was to demonstrate this effect of Endostar using A549 and NCI-H1299 lung cancer cells.

## Materials and methods

### Culture and preparation of lung cancer cells

Human A549 and NCI-H1299 lung cancer cells were maintained in standard cell culture conditions. The cells whose density were 9 × 10^5^/well were seeded in 6-well culture plates for 24 hours culture to settle. The temperature was 37 °C. The atmosphere was humidified and contained 5% CO2 in F-12K (A549 cells) or RPMI-1640 (NCI-H1299 cells) medium supplemented with 10% FBS. Then, the cells were precultured for 24 h in the medium containing 1% FBS, followed by the addition of Endostar (a Modified Recombinant Human Endostatin expressed and purified in E. coli, which was purchased from Simcere Pharmaceutical Research Co., Ltd., Nanjing, China) into the wells to the indicated final concentration (6.25 μg/ml, 12.5 μg/ml, and 25 μg/ml with 0 μg/ml as control) for a further 24 h incubation. Cells were collected for analysis. All experiments were individually carried out three times. This study was approved by the Ethics Committee of the Affiliated Hospital of Chengde Medical University in biomedicine (CYFYLL2021091).

### Transfection of HIF-1 into cells

To overexpress HIF-1, the cells were transiently transfected with Human HIF1A ORF mammalian expression plasmid (Sino Biological Inc., Beijing, China) using Lipofectamine™ LTX Reagent with PLUS™ Reagent (Invitrogen, CA, USA), according to the manufacturer’s instructions. Forty-eight hours after transfection, the cells were harvested for assays.

### Immunocytochemistry

The Endostar treated and untreated cells grown on glass slides were washed and fixed using cold methanol for 5 min. Next, in order to inhibit the endogenous peroxidase activity, cells were treated with 3% hydrogen peroxide in methanol. Then, in order to avoid nonspecific binding, cells were blocked with 10% normal serum. After overnight incubation at 4 °C with goat polyclonal primary antibody against HIF-1 (Affinity biosciences, Suzhou, China) (at a 1:100 dilution), the cells were hybridized by corresponding secondary antibody for 1 h, and finalized with a diaminobenzidine solution to detect the target antigen. The nucleus of cells was counterstained by hematoxylin before mounting. A light microscope was used to examine the slides.

### Western blot

After extraction of the total cellular proteins from the Endostar treated and untreated cells grown in 6-well culture plates by lysis with radioimmunoprecipitation assay (RIPA) buffer (Beijing Solarbio Science & Technology Co., Ltd., Beijing, China) and quantification with BCA Protein Assay Kit (Beijing Solarbio Science & Technology Co., Ltd., Beijing, China), 30 μg of the proteins, diluted in loading buffer and denaturized for 5 minutes at the temperature of 100 °C following keeping on ice for 10 minutes, were subjected to 10% (for HIF1, MHC-1, STAT3, and pSTAT3) or 12% (for β2 m) sodium dodecyl sulfate-polyacrylamide gel electrophoresis (SDS-PAGE). The proteins were then electrotransferred onto polyvinylidene fluoride (PVDF) membrane, blocked for 1 hour with 5% skimmed milk at room temperature, before overnight probe at the temperature of 4 °C using relevant primary antibodies, polyclonal rabbit anti-HIF-1 antibody (Affinity biosciences, Suzhou, China), recombinat monoconal rabbit anti-β2 m antibody (HuaBio Inc., Cambridge, USA), recombinat monoconal rabbit anti-HLA-ABC antibody (HuaBio Inc., Cambridge, USA), recombinat monoconal rabbit anti-STAT3 and anti-pSTAT3 antibody (HuaBio Inc., Cambridge, USA), and mouse monoclonal anti-β-actin antibody (Proteintech, Rosemont, Illinois, USA) following 2-h incubation with horseradish peroxidase-conjugated secondary antibodies. The protein bands were visualized using enhanced chemiluminescence (ELC) detection system (Amersham, Arlington Heights, IL, USA). The expression of β-actin serves as an endogenous control. The acquired images were quantified with normalizative to β-actin using ImageJ software (Rasband, W.S., ImageJ, U.S. National Institutes of Health, Bethesda, Maryland, USA).

### Quantitative real-time PCR

Total RNAs in Endostar treated and untreated cells grown in 6-well culture were extracted by using Superbrilliant® 6 min High-quality RNA Extraction Kit (Zhongshi Gene Technology, Tianjin, China) under Rnase free condition according to the protocol of manufacturer. Fast Quant RTkit (Tiangen Biotech Co., Ltd., Beijing, China) was utilized to synthesize cDNAs. Quantitative real-time PCR (qPCR) was conducted on Roche Cobas z 480 Real-Time PCR Detection System (Roche, Basel, Switzerland) using SuperReal PreMix Plus kit (Tiangen Biotech Co., Ltd., Beijing, China), and the responsible primers, β2 m (Invitrogen, Carlsbad, USA), HLA-ABC α heavy chain (Invitrogen, Carlsbad, USA), and β-actin (Thermo Fisher Scientific Inc., Waltham, Massachusetts, USA), were used. The expression of β-actin mRNA as a control was used for normalization of the mRNAs expression with 2^−ΔΔCt^ method to calculate the fold changes of the mRNAs.

### Statistical analysis

The data were described as mean ± standard deviation (SD) and the statistical significance was determined by one-way ANOVA before LSD test for multiple comparisons and independent-sample t-test for comparison between the two groups. The level of *p* < .05 was used to confirm that the differences were statistically significant.

## Results

### Inhibition on the expression of HIF-1

**Figure 1.**
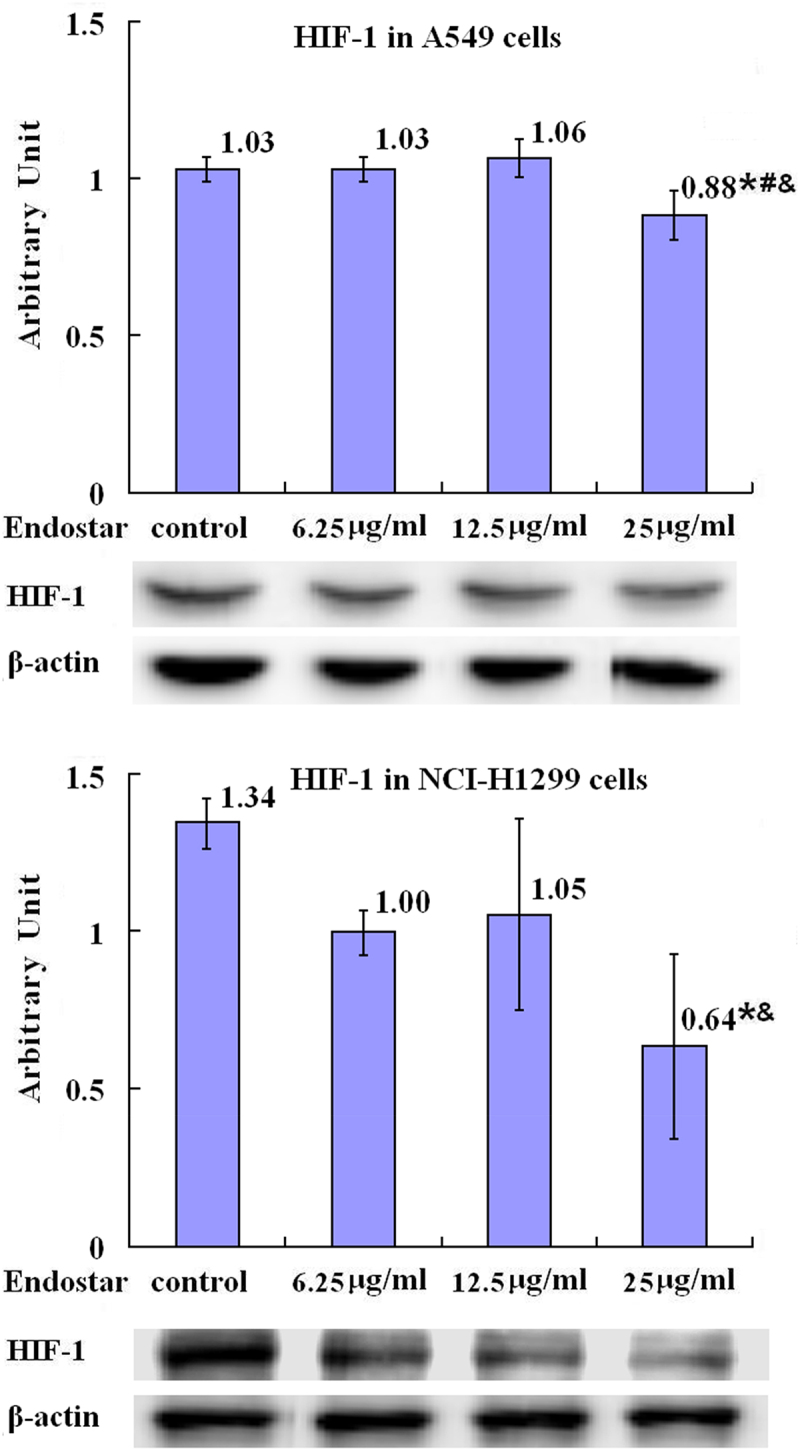
Inhibition by Endostar on HIF-1 in A549 and NCI-H1299 lung cancer cells measured by Western blot assay. After treatment with Endostar for 24 h, the HIF-1 in A549 and NCI-H1299 lung cancer cells was measured by Western blot assay. Error bars indicate the SD. Asterisks indicate *p* < .05, significantly different compared with the control; Hashtags indicate *p* < .05, significantly different compared with the group treated with 6.25 μg/ml Endostar; ampersands indicate *p* < .05, significantly different compared with the group treated with 12.5 μg/ml Endostar; one-way analysis of variance followed by LSD test.

**Figure 2.**
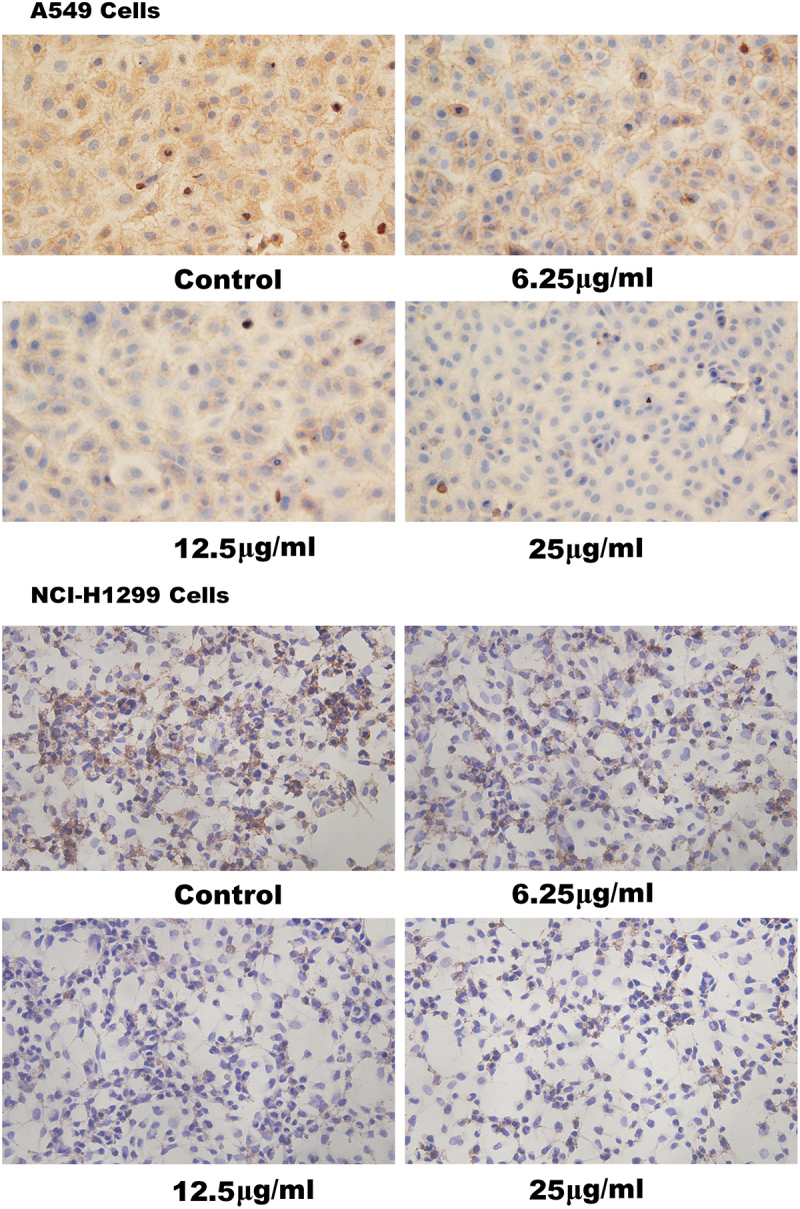
Inhibition by Endostar on HIF-1 in A549 and NCI-H1299 lung cancer cells measured by immunocytochemistry. After treatment with Endostar for 24 h, the HIF-1 in A549 and NCI-H1299 lung cancer cells was measured by immunocytochemistry. Photographs were taken under microscope (400× magnification).

It was shown with western blot assays that, in A549 lung cancer cells, treatment with Endostar for 24 h decreased the levels of HIF-1 protein in the groups treated with 25 μg/ml Endostar with statistical significance compared with the control group (*p* = .015), 6.25 μg/ml group (*p* = .014) and 12.5 μg/ml group (*p* = .005), and in NCI-H1299 lung cancer cells, treatment with Endostar decreased the levels of HIF-1 protein in the groups treated with 25 μg/ml Endostar with statistical significance compared with the control group (*p* = .004) and 12.5 μg/ml group (*p* = .047) (Figure 1 in detail), which was similar to immunocytochemistry showing the reduction of HIF-1 protein in Endostar treated A549 and NCI-H1299 lung cancer cells compared with the control cells (Figure 2).

### Promotion on the expression of β2-microglobulin

**Figure 3.**
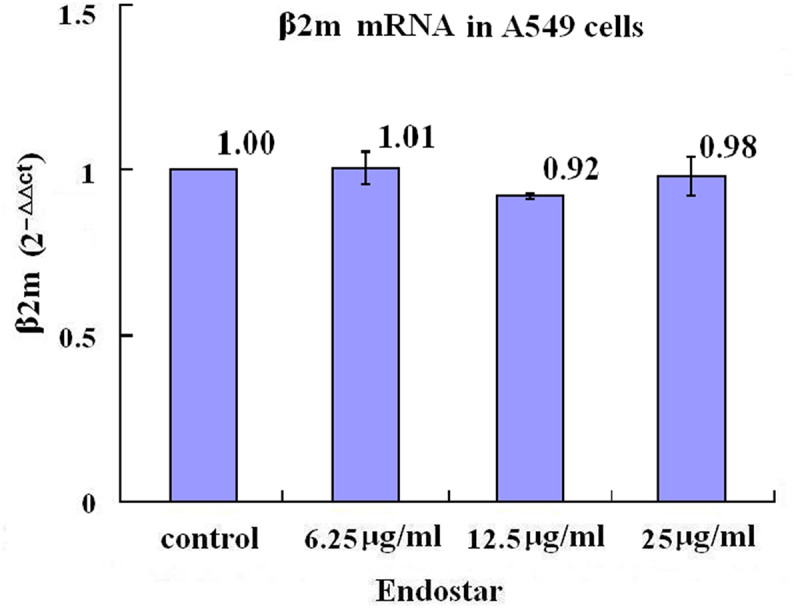
Effects of Endostar on β2-microglobulin mRNA in A549 lung cancer cells measured by RT-qPCR assay. After treatment with Endostar for 24 h, the β2-microglobulin mRNA in A549 lung cancer cells was measured by RT-qPCR assay. Error bars indicate the SD. No significant differences among the groups were found (*p* > .05); one-way analysis of variance.

**Figure 4.**
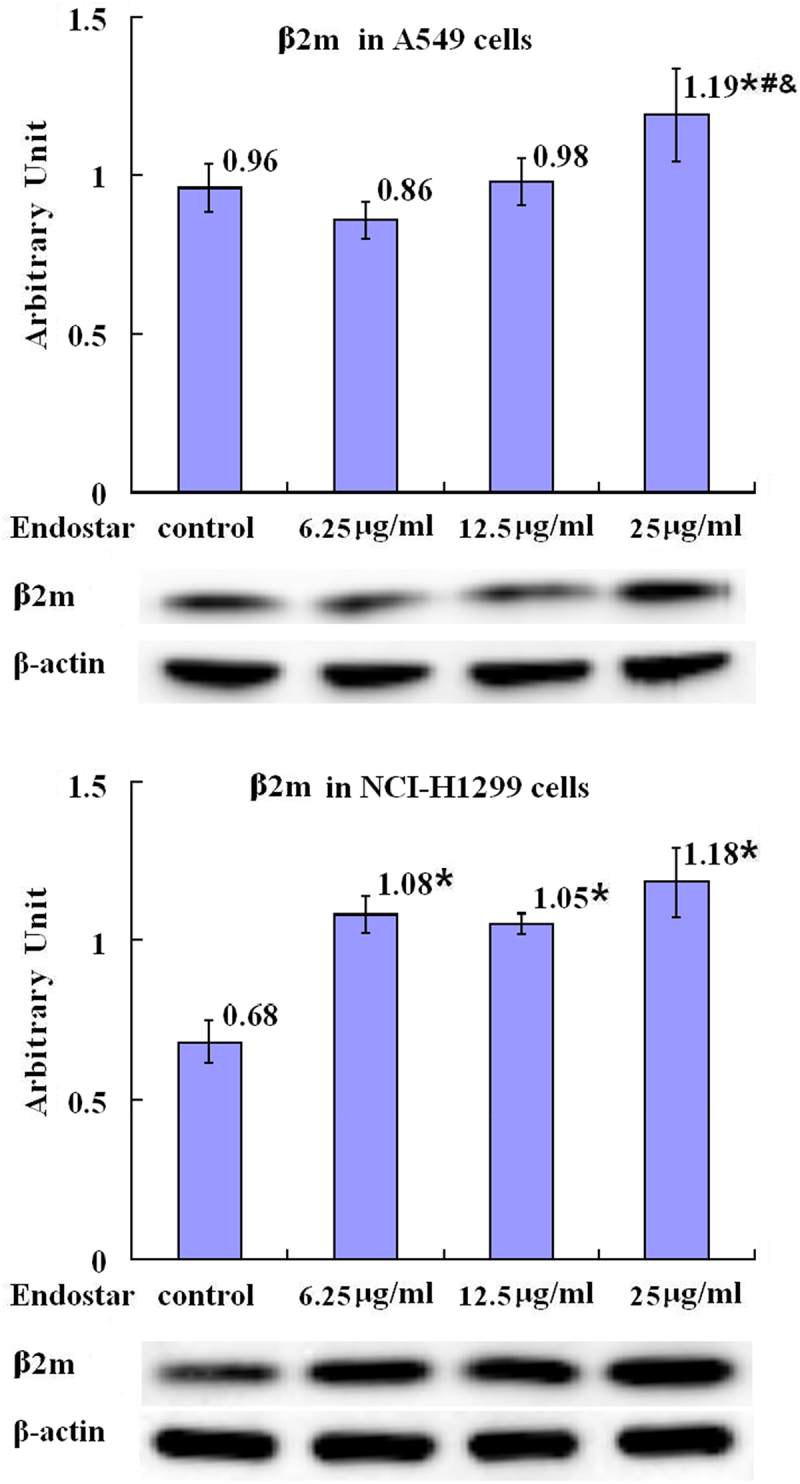
Upregulation by Endostar on β2-microglobulin protein in A549 and NCI-H1299 lung cancer cells measured by Western blot assay. After treatment with Endostar for 24 h, the β2-microglobulin protein in A549 and NCI-H1299 lung cancer cells was measured by Western blot assay. Error bars indicate the SD. Asterisks indicate *p* < .05, significantly different compared with the control; Hashtags indicate *p* < .05, significantly different compared with the group treated with 6.25 μg/ml Endostar; ampersands indicate *p* < .05, significantly different compared with the group treated with 12.5 μg/ml Endostar; one-way analysis of variance followed by LSD test.

Beta 2-microglobulin (β2 m) is the invariable light chain of the MHC class I heterodimer molecules and associated with MHC class I down-regulation which is possibly caused by high level of HIF-1. It was shown in this study that with the reduction of the HIF-1 level, unlike the β2 m mRNA levels without statistically significant differences among the Endostar treated and untreated groups (*p* = .098) according to RT-qPCR assay (Figure 3 in detail), the higher relative levels of β2 m protein were found in the A549 cells in the group which were treated with 25 μg/ml Endostar with statistical significance compared with the control group, 6.25 μg/ml group, and 12.5 μg/ml group (*p* = .017, *p* = .003, *p* = .026, respectively), indicating that the effect of Endostar on the β2 m expression in A549 cells may be mRNA independent, and in the NCI-H1299 cells in the groups which were treated with 6.25 μg/ml, 12.5 μg/ml, and 25 μg/ml (*p* = .000, *p* = .000, *p* = .000, respectively) Endostar, with statistical significance compared with the control group (Figure 4 in detail).

### Promotion on the expression of MHC class I (HLA-ABC) α heavy chain

**Figure 5.**
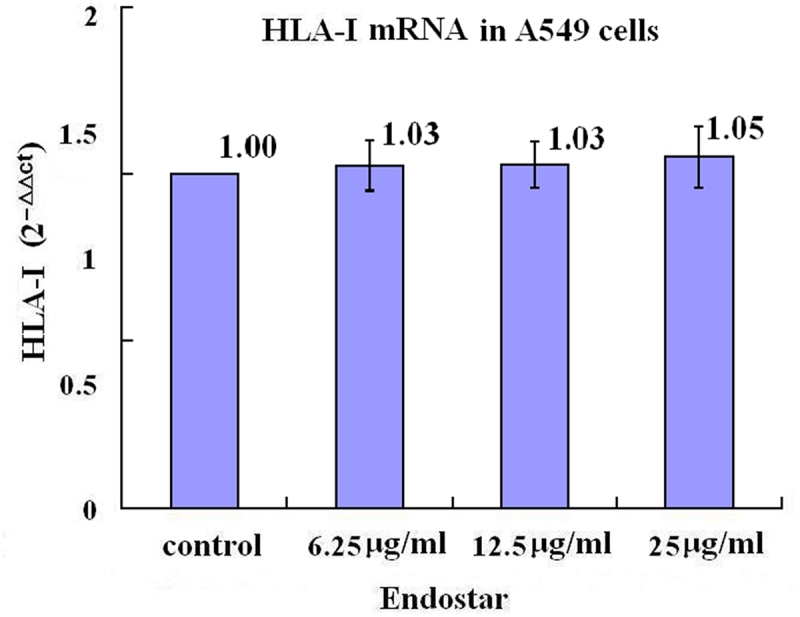
Effects of Endostar on the mRNA of MHC class I α-heavy chain in A549 lung cancer cells measured by RT-qPCR assay. After treatment with Endostar for 24 h, the mRNA of MHC class I α-heavy chain in A549 lung cancer cells was measured by RT-qPCR assay. Error bars indicate the SD. Slight enhancement without statistical significance were found among the groups with Endostar treatment (*p* > .05); one-way analysis of variance.

**Figure 6.**
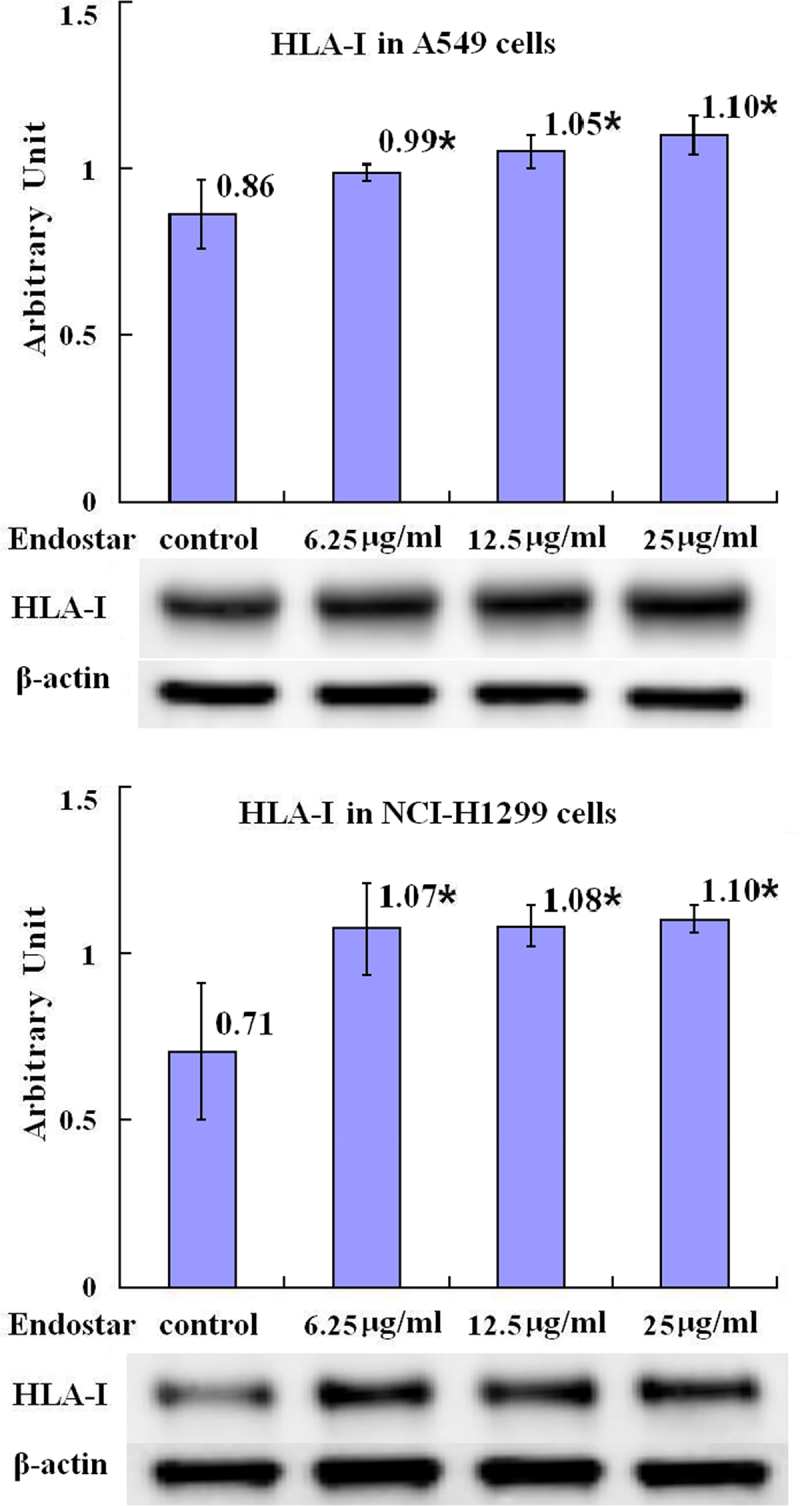
Upregulation by Endostar on the protein of MHC class I α-heavy chain in A549 and NCI-H1299 lung cancer cells measured by Western blot assay. After treatment with Endostar for 24 h, the protein of MHC class I α-heavy chain in A549 and NCI-H1299 lung cancer cells was measured by Western blot assay. Error bars indicate the SD. Asterisks indicate *p* < .05, significantly different compared with the control; one-way analysis of variance followed by LSD test.

The loss of MHC class I expression was probably caused by high level of HIF-1. After a treatment of A549 lung cancer cells with Endostar for 24 hours, the relative levels of MHC-I α heavy chain mRNA demonstrated a slight enhancement without statistical significance (*p* = .840) in the groups treated with 6.25 μg/ml, 12.5 μg/ml, and 25 μg/ml Endostar compared with the control group (Figure 5 in detail). The relative levels of MHC-I protein α heavy chain in the A549 cells in the groups treated with 6.25 μg/ml, 12.5 μg/ml, and 25 μg/ml Endostar were statistically enhanced (*p* = .048, *p* = .007, *p* = .002, respectively) compared with the control group, and in the NCI-H1299 cells in the groups treated with 6.25 μg/ml, 12.5 μg/ml and 25 μg/ml Endostar were statistically enhanced as well (*p* = .008, *p* = .007, *p* = .005, respectively) compared with the control group (Figure 6 in detail).

### Downregulation on the expression of β2-microglobulin and MHC-I α heavy chain by over-expression of HIF-1

**Figure 7.**
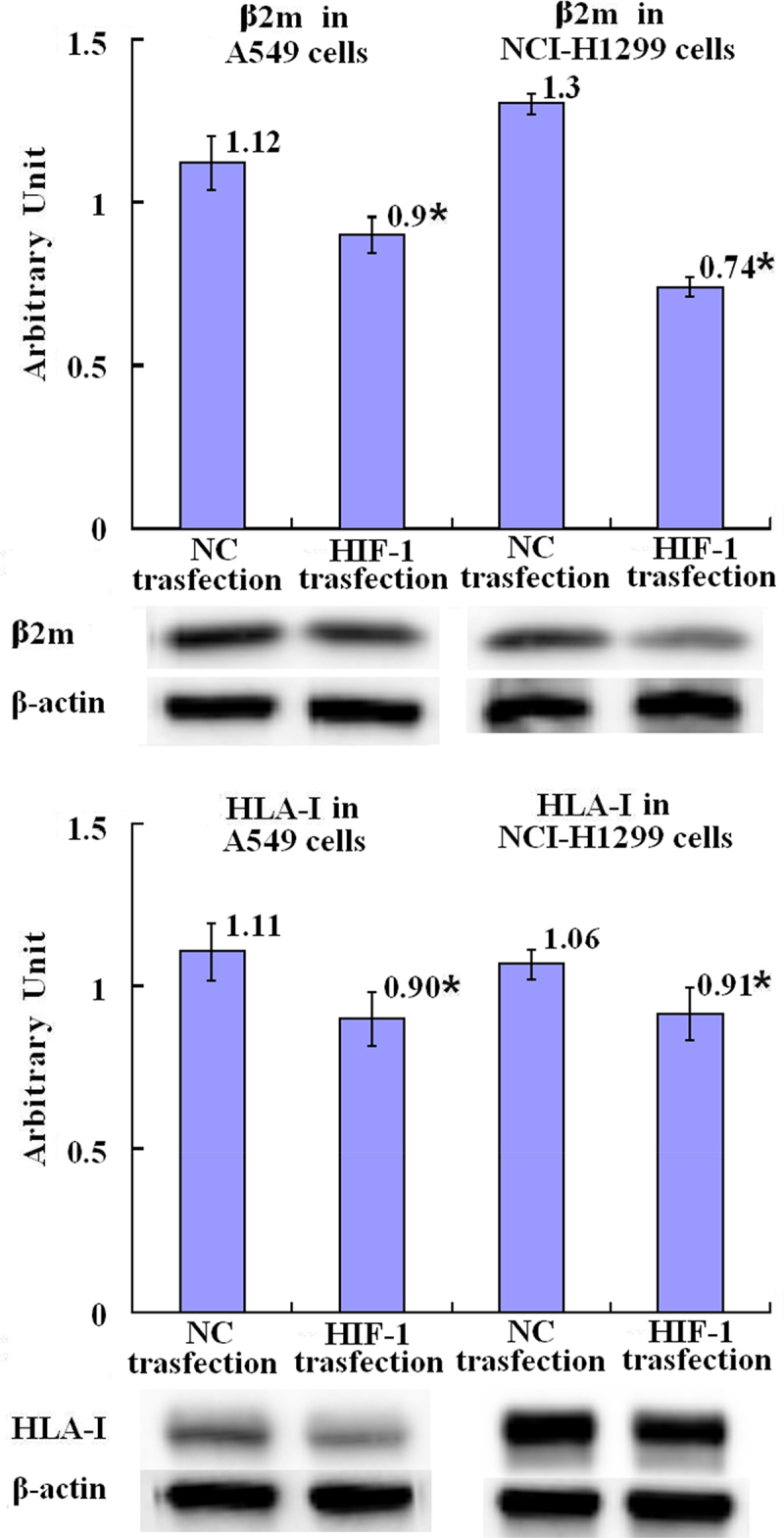
Downregulation by HIF-1 over-expression on β2-microglobulin and MHC class I α-heavy chain in A549 and NCI-H1299 lung cancer cells measured by Western blot assay. After transfection with over-expressing HIF-1 gen into the cells for 48 h, the proteins of β2-microglobulin and MHC class I α-heavy chain in A549 and NCI-H1299 lung cancer cells were measured by Western blot assay. Error bars indicate the SD. Asterisks indicate *p* < .05, significantly different compared with the control; independent-sample t test.

HIF-1 gene was transfected into A549 and NCI-H1299 cells to overexpress HIF-1. It was shown that the relative levels of β2 m (β light chain) and α heavy chain of MHC-I protein were statistically decreased in HIF-1-over-expressing A549 cells and NCI-H1299 cells compared with the NC control A549 cells and NCI-H1299 cells (*p* = .019, *p* = .041, *p* = .000, and *p* = .048), respectively (Figure 7 in detail).

### Downregulation on the expression of STAT3 and pSTAT3

**Figure 8.**
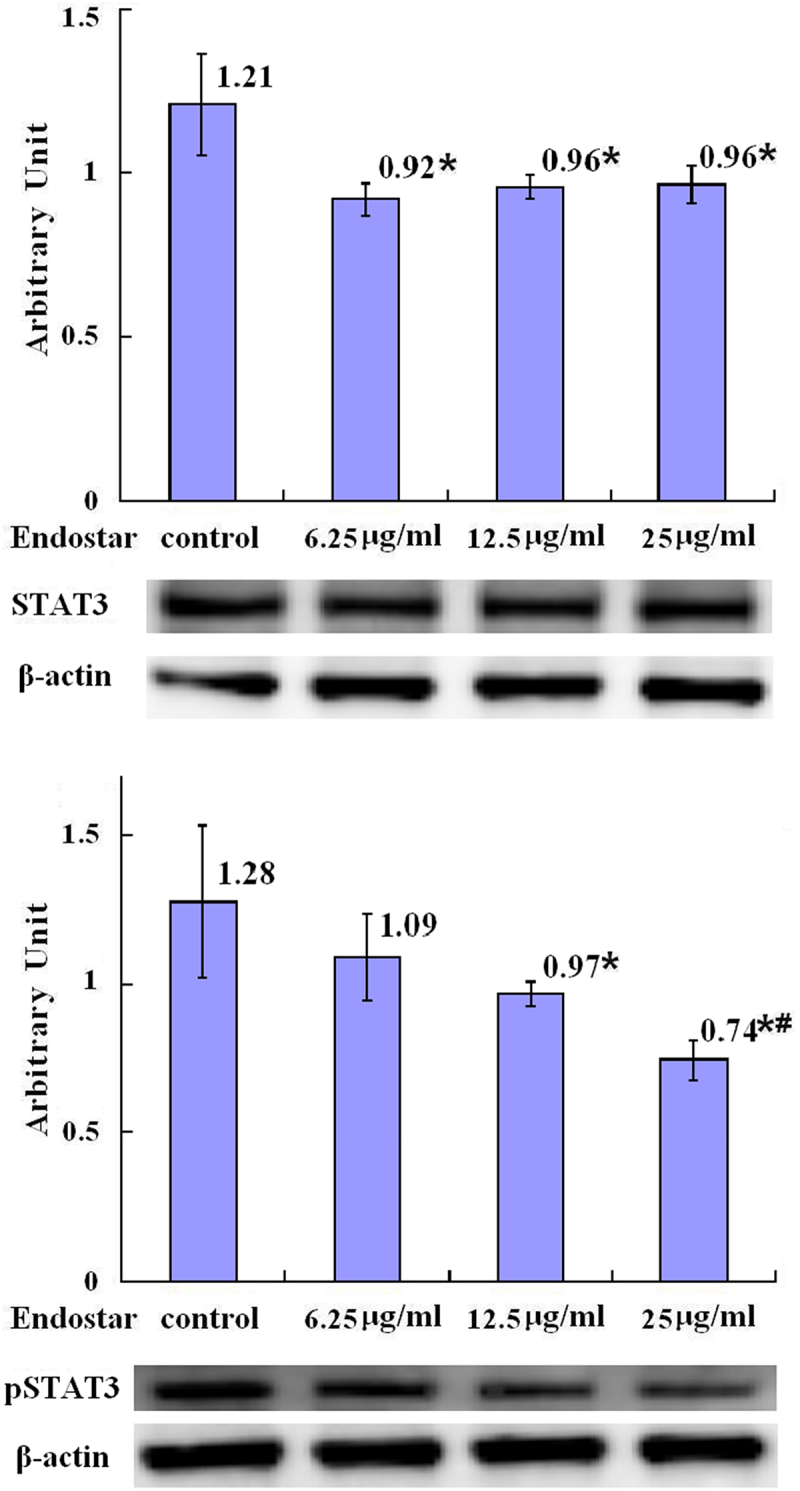
The downregulation of STAT3 and pSTAT3 proteins in A549 lung cancer cells by Endostar measured by Western blot assay.After treatment with endostar for 24 h, the protein of STAT3 and pSTAT3 in A549 lung cancer cells was measured by Western blot assay. Error bars indicate the SD. Asterisks indicate *p* < .05, significantly different compared with the control; Hashtags indicate *p* < .05, significantly different compared with the group treated with 6.25 μg/ml Endostar; one-way analysis of variance followed by LSD test.

MHC class I may be regulated by JAK2/STAT3 signaling pathway. It was shown that the relative levels of STAT3 in A549 cells in the groups treated with 6.25 μg/ml, 12.5 μg/ml, and 25 μg/ml Endostar were statistically decreased (*p* = .004, *p* = .008, *p* = .010, respectively) compared with the control group (Figure 8 in detail). STAT3 is activated by phosphorylation. After a treatment of A549 lung cancer cells with Endostar for 24 hours, the relative levels of pSTAT3 were statistically decreased with statistical significance in the groups treated with 12.5 μg/ml (*p* = .037) and 25 μg/ml (*p* = .003) Endostar compared with the control group (Figure 8 in detail).

## Discussion

The host immune system exerts immunosurveillance to control cancer development. By inducing the expression of immunosuppressive factors, however, tumor HIF-1 signaling subdues both the innate and adaptive immune responses, thereby shielding the tumor from immune attacks. In addition, insufficient expression of MHC class I on cancer cells is one of the important strategies employed by cancer to evade the immune system. Therefore, inhibition of HIF-1 and improvement of MHC class I expression on cancer cells is important to induce effective responses of cell-mediated immunity against cancer such as lung cancer for immunotherapies, including PD-1/PD-L1 inhibitor. The effect of Endostar in inhibiting HIF-1 and promoting MHC class I expression on cancer cells, such as lung cancer cells, may counteract cancer immune evasion and thereby benefit cancer immunotherapy. However, this remains unclear. This study was designed to determine the effect of Endostar in inhibiting HIF-1 and promoting MHC class I expression on lung cancer cells. Endostar was administrated to A549 and NCI-H1299 lung cancer cells and the protein of HIF-1 and MHC class I and their mRNAs was detected by western blot and RT-qPCR. The lower HIF-1 and higher MHC class I were found in Endostar treated A549 and NCI-H1299 cells. These findings demonstrated inhibtion on HIF-1 and promotion on MHC class I by Endostar, suggesting the potential of Endostar to benefit lung cancer immunotherapy.

Endostar, an N-terminal modified recombinant human endostatin, is a human recombinant endostatin, an attractive anti-angiogenesis protein. Therefore, Endostar has anti-angiogenesis effects. Endostar was developed as a specific drug permitted by the State Food and Drug Administration in China in 2005 for its use in NSCLC therapy.^25,26^ Endostatin is a natural protein, first isolated and extracted from mouse tumors by Judah Folkman, with a wide antitumor spectrum and strong antiangiogenic capacity.^27,28^ Angiogenesis is a physiological process of forming new blood vessels from existing blood vessels and circulating endothelial precursors. It is essential to the occurrence and development of tumors, which grow rapidly and metastasize eventually.^24^ Physiologically, angiogenesis is essential to physiological processes like embryogenesis, tissue growth, and regeneration.^29,30^ Oncologically, it is also important for cancer cells that grow rapidly and metastasize eventually because angiogenesis supplies oxygen and nutrients which are deficient in cancer cells for their rapid growth and eventual metastasis.^31^ Therefore, anti-angiogenesis is one of the most important cancer therapies.^32^ Endostar as a recombinant human endostatin with nine added amino acids (MGGSHHHHH)^33^ to maintain stability and a long half-life has effective antiangiogenic effect. Besides, it was shown in this study that Endostar inhibited HIF-1 with upregulation of MHC class I expression in A549 and NCI-H1299 lung cancer cells, benefiting cancer cell killing by effectory lymphocytes.

HIF-1, a heterodimer consisting of a constitutive β-subunit and an oxygen-sensitive α-subunit,^34^ is a main transcriptional regulator responsible for metabolic adaptation to alterations in the oxygen environment. It involves in many physiological and pathological processes in the body and is closely associated with the pathogenesis of many diseases.^13^ In solid tumors, uncontrolled proliferation of cancer cells vs disorganized growth of blood vessels results in limited supply of nutrients and oxygen resulting in low oxygen tension; therefore, regions with hypoxic microenvironments are created. In these regions, the highly overexpressed HIF-1 is important to drive tumor growing, invasion, and metastasis in different human cancers.^35,36^ The association of a poor survival with the high expression of HIF-1α has been indicated by survival analysis in patients with lung cancer, and different SNPs in HIF-1α may have different effects on overall cancer risk in an ethnicity- and type-specific manner.^37^ Reduction of HIF-1α by Simvastatin enhanced Anti-tumor Effects of Bevacizumab in A549 cells.^38^ Targeting ATM/HIF-1α signaling by solanidine induced anti-angiogenesis and anti-cancer effect in lung cancer.^39^ Besides its involvement in various aspects of tumor development, such as tumor growth, invasion, metastasis, and angiogenesis, HIF-1 is also involved in tumor immune evasion which facilitates cancer cells to proliferate and metastasize, and contributes to failure in immunotherapy.^40^ Inhibition of HIF-1 expression in cancer cells contributes to cancer control and induction of effective anti-cancer immunity in cancer immunotherapy. Endostar has the effect to down-regulate HIF-1.^41^ It was shown in this study that 25 μg/ml Endostar inhibited HIF-1 expression in A549 and NCI-H1299 lung cancer cells.

HIF-1 is induced by hypoxia in cancer cells,^42^ and in normoxia, it is not usually observed or only basal expression can be observed.^43,44^ In this study, experiments showed a high expression of HIF-1 in control and untreated cells. However, this did not necessarily mean that the expression of HIF-1 in control and untreated cells was really high because it was detected with the expression in Endostar treated A549 cells and NCI-H1299 cells as the backgroud. The expression detected by Western blot and Immunocytochemistry is relative quantification. The assays had been optimized for enough sensitivity to detect the reduced expression in Endostar treated A549 cells and NCI-H1299 cells.

An effective adaptive response can be achieved through a multi-step antigen processing and pathway, namely the cellular antigen processing machinery (APM). Antigens must be processed into antigenic peptides by APM. These peptides are loaded onto an MHC class I molecule. MHC class I molecules are glycoproteins of heterodimers with a polymorphic heavy chain (α-chain) and an invariable β2 microglobulin (β2 m) light chain (β-chain). The α-chain is encoded by the Human Leukocyte Antigen-HLA A, B, and C genes in humans. There is a groove in the MHC class I molecules to preferentially bind 8-11mer peptides. The antigenic epitope binds to the exposed surface of this groove as a part of the MHC class I complex. It is recognized by the TCR on the T cells.^45^ This study showed that the expression of MHC class I α-heavy chain and β2 m light chain in A549 and NCI-H1299 lung cancer cells was improved by Endostar, which benefited cancer immunotherapy against the lung cancer cells.

In the context of HIF-1 down-regulation by Endostar, which was shown in this study, to demonstrate the role of HIF-1 on the MHC class I α-heavy chain and β2 m light chain in A549 and NCI-H1299 lung cancer cells, the over-expressing HIF-1 gene was transfected into A549 and NCI-H1299 cells resulting in decrease of relative levels of MHC class I α-heavy chain and β2 m light chain. This results were in line with the decrease of HIF-1 by Endostar treatment accompanied with the enhancement of MHC class I α-heavy chain and β2 m light chain.

In addition to the important role in metabolic adaptation to hypoxia stress caused by deficient supply of nutrients and oxygen because of uncontrolled growth of cancer cells and disorganized neoangiogenesis, the signaling pathways of HIF-1, a heterodimer highly expressed in a variety of tumor cells, suppress innate and adaptive immune systems to escape immune attack. That HIF-1 downregulates the antigen presenting MHC class I molecules is an important strategy for cancer to evade immune attack, because only in combination with MHC class I on the target cells, can tumor antigenic peptides be recognized by CD8+ CTL with the subsequent destruction of the target cells. Theoretically, inhibition of HIF-1 may upregulate the expression of MHC class I on cancer cells. This study demonstrated that 25 μg/ml Endostar inhibited expression of HIF-1 with the upregulation of MHC class I α-heavy chain and β2 m light chain in A549 and NCI-H1299 lung cancer cells, suggesting the potential for Endostar to facilitate cancer immunotherapy. Experimental evidence, however, is required and further research with experimental evidence remains to be performed in the future to confirm the possible suppression of immune evasion tactic with endostar treatment. Other limitations such as lack of an in vivo tumor model and further validation using flow cytometry remain to be supplementarily performed in the future.

The mechanism of MHC class I regulation involves the innate immune molecule NLR family CARD domain containing 5 (NLRC5), which is a member of recently discovered NLRs-like receptor family of the highly conserved one.^46^ NLRC5 is an MHC class I gene transactivation factor which induces the MHC class I gene transcription and subsequently activates antigen presentation process.^47,48^ NLRC5 is transcriptionally regulated in JAK2/STAT3 signaling-dependent pathway.^49^ This study demonstrated that some concentrations of Endostar decreased the relative levels of STAT3 and pSTAT3 in A549 lung cancer cells with statistical significance, which is in line with the role of JAK2/STAT3 pathway in regulation of MHC class I via NLRC5, suggesting the underlying mechanism of MHC class I upregulation involving JAK2/STAT3 signaling pathway.

## Conclusions

Endostar (25 μg/ml) inhibited the expression of HIF-1 with the upregulation of MHC class I α-heavy chain and β2 m light chain in A549 and NCI-H1299 lung cancer cells, which showed the potential for Endostar to facilitate cancer immunotherapy. It is needed for future studies that are warranted to confirm the role of Endostar treatment.

## Data Availability

All the data generated or analyzed during this study are included in this published article.
